# Does testing enhance learning in continuing medical education?

**Published:** 2018-07-27

**Authors:** Meghan McConnell, Chenchen Hou, Mohamed Panju, Akbar Panju, Khalid Azzam

**Affiliations:** 1 Department of Innovation in Medical Education, University of Ottawa, Ontario, Canada; 2 Department of Anesthesiology and Pain Medicine, University of Ottawa, Ontario, Canada; 3 McMaster University, Ontario, Canada

## Abstract

**Background:**

There has been growing interest in using theory-driven research to develop and evaluate continuing medical education (CME) activities. Within health professions education, testing has been shown to promote learning in a variety of different contexts, an effect referred to as test-enhanced learning (TEL). However, the extent to which TEL generalizes to CME remains unclear. The purpose of this study was to investigate whether physicians who received two intervening tests following a CME event would experience a TEL effect relative to physicians who received additional study material to review without testing.

**Methods:**

Forty-nine physicians were recruited during a local CME activity. Physicians were randomized to either a) the test group (n=26), where participants completed two 20 multiple-choice question (MCQ) quizzes related to the lecture content or b) the study group (n=23), where participants studied the same information without testing. Testing and studying occurred independently during the CME activity, and then four weeks later online. At eight weeks, participants completed a final 20-item MCQ online test. A between-subjects *t*-test was used to compare performance on the final test as a function of the initial educational activity (test group vs. study group).

**Results:**

Performance on the final MCQ test was equivalent for both test (Mean (SD): 75% (9.9)) and study-only (77% (7.3)) conditions (t(47) = 0.94, p=0.35).

**Conclusion:**

The null findings in the present study are contrary to previous findings demonstrating TEL among novice learner populations. The lack of TEL highlights several programmatic considerations that should be factored in before implementing TEL as a part of CME.

## Introduction

As part of their professional responsibilities, clinicians are expected to engage in various continuing medical education (CME) activities geared toward maintaining and/or improving competence within their clinical and professional roles.^1^ Researchers have emphasized the need for theoretically-driven research to evaluate and improve the educational value of CME activities.^2^

Relative to studying, testing is associated with enhanced memory of the same information, an effect referred to as “test-enhanced learning” (TEL).^3-5^ TEL has been well established in authentic educational contexts; for example, within medical education, testing has been shown to improve long-term retention in both undergraduate^6-8^ and postgraduate^9^ trainees. Such research suggests that testing can serve as a relatively inexpensive way to enhance long-term memory of relevant information.^10,11^

Why does testing enhance learning? The leading theory emphasizes the importance of memory retrieval processes in facilitating learning.^5^ Simply put, the act of taking a test requires individuals to actively retrieve information from memory, thus strengthening retrieval pathways in memory and making it easier to retrieve this information in the future.

However, nearly all TEL studies have been conducted on individuals who are in the process of developing foundational knowledge and skills. If TEL improves learning by developing retrieval pathways in memory, the effects may not be transferable to CME contexts, as practicing clinicians have plenty of practice retrieving relevant information in authentic clinical contexts. The few studies that have investigated TEL within CME have reported inconsistent results. For example, McConnell et al. found that physicians who completed a short answer test following a large group passive CME activity did not remember any more information on a test four weeks later than physicians who studied the same information.^12^ In contrast, Larsen et al.^13^ found that within a CME context, physicians who received weekly short-answer quizzes performed better on a final test five and a half months later, relative to those who repeatedly studied the same information.

One major difference between these two studies is the number of intervening tests provided to CME participants; Larsen et al.^13^ provided participants with four intervening quiz/study activities prior to the final test, while McConnell et al.^12^ only provided a single quiz/study activity. Research shows that increasing the number of intervening tests enhances TEL, provided the tests are separated by a long enough period (e.g., days, weeks) to require effortful memory retrieval.^14^ However, within CME, there are clear logistical challenges with increasing the number of test activities. Practicing clinicians have an aversion towards taking tests, and when combined with busy clinical duties, this may result in loss of participants across test activities. With this in mind, the present study examined whether TEL would be observed for physicians who received two intervening tests following a CME event, relative to physicians who received matched study activities.

## Methods

The research project was approved by the Hamilton Integrated Research Ethics Board (#14-255).

### Participants

The study took place during a 3-day Review Course in Internal Medicine at McMaster University. The target audience included general internists, subspecialists, ER physicians, family physicians and general practitioners. During the CME event, 75 physicians consented to participate, 40 of which were randomized to the test group while 35 were randomized to the study group. Of these 75 participants, 55 (70%) participated in the second activity, 29 from the test condition and 26 from the study condition. Forty-nine (65%) physicians completed the final test activity (26 from the test condition and 23 from the study condition). Despite substantial attrition across the educational activities, the drop-out rate was nearly identical across the two groups. Participants who completed the entire study received a $50 gift card in appreciation for their participation.

A priori power analyses were conducted to determine an appropriate sample size for this study. A recent meta-analysis^15^ on TEL reported the mean effect size related to testing ranged from moderate (d=0.55) to large (d=0.88). Using the smaller effect size, 22 participants/group would be required to detect a difference with a power of 80% and a significance level of 0.05, which we achieved even after attrition.

### Materials

Educational materials were based on the contents of four CME courses: “Chronic Angina,” “Acute Coronary Syndrome,” “Smoking Cessation,” and “Dyslipidemia.” Materials were developed by a panel of experts consisting of the CME course instructors and members of the research team (CH, KA). For each course, the panel identified five learning objectives that would be taught to participants. This resulted in a total of 20 learning objectives (five learning objectives * four courses) which served as the blueprint for the construction of interventional (e.g., quiz/study materials) and final assessment activities.

#### Quizzes

Each intervening quiz consisted of 20 multiple choice questions (MCQs), with each MCQ relating to a specific learning objective. MCQs consisted of short clinical vignettes with a lead-in question,^16,17^ followed by four response options, with only one being correct. For each learning objective, the panel created two MCQs. These MCQs were matched to the same learning objective but had slightly different clinical vignettes (e.g., 55 yo male plumber vs. 59 yo male electrician). This allowed us to generate two quizzes that were blueprinted to the same learning objectives but differed in irrelevant patient characteristics.

#### Study material

Study materials were simply reading materials, much the same as other TEL studies within medical education.^6,12,13^ For each learning objective, a study item was created to provide participants in the control group with the same information as the test group, but in a format that did not require retrieval of information from memory, just reading.

#### Final assessment

The final test consisted of 20 new MCQs that were generated in the same way as the intervening quizzes. Each MCQ consisted of a short clinical vignette with a lead-in question that was matched to the same 20 learning objectives, which ensured that the final test was blueprinted to the same content as the quiz/study materials.

### Research Methods

Learning materials were developed from four sessions (“Chronic Angina,” “Acute Coronary Syndrome,” “Smoking Cessation,” and “Dyslipidemia”) that were held on the first day of the three-day CME event.

On the third day, the first quiz/study activity took place in a separate room during lunchtime. Upon entering the room, participants were handed an envelope that contained paper copies of either a 20-item MCQ test (test condition) or a study handout (study condition). Members of the research team were blinded to the contents of the envelope. Participants in the test condition were asked to complete the test independently without using external resources and were not given any feedback on their performance. Those in the study condition were asked to read the contents on the handout independently. Upon completion, participants returned all study materials (e.g., quizzes/study sheets).

Four-weeks later, participants were sent emails with a link to the second phase of the study. Those in the test group completed an online quiz consisting of 20 new MCQs, matched in content. Participants in the control group viewed an online study handout that was identical to that provided during the first study activity (e.g., same handout, but provided online). Again, participants were asked to complete the activities independently without using external resources. Participants in the test condition did not receive feedback.

The final phase of the study took place eight weeks after the initial CME event. During this phase, all participants completed a final online test composed of 20 new MCQs matched in content from initial educational activities.

### Data analysis

A between-subjects *t*-test was used to compare performance on the final test across the two groups (e.g., test vs. study). The dependent variable was the mean proportion of correct answers on the final test activity.

## Results

**Figure 1:**
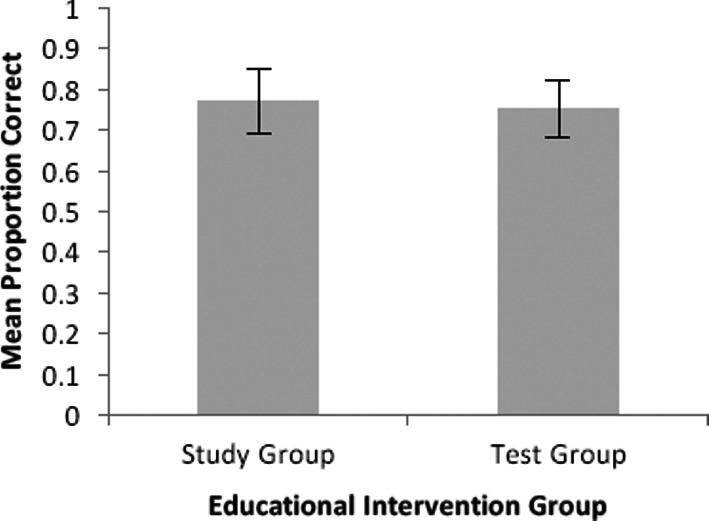
Mean proportion correct score on the final quiz as a function of educational activity. Error bars represent standard error of the group mean.

We did not find a significant effect of testing (*t*(47)=.94, *p* =.35). As seen in Figure 1, performance on the final test did not differ significantly between physicians in the test group (*M*=0.75, *SD*=1.0) and the study group (*M*=0.77, *SD*=0.7).

## Discussion

This study examined TEL among practising physicians in the context of a local CME event. Our study did not find a benefit of testing: clinicians who received two intervening tests performed equivalently on a final test relative to those who studied the same material. One potential interpretation of these data is that TEL may not benefit practicing physicians as much as other learner populations. However, we caution against this interpretation, especially in light of a recent study by Larsen et al.,^13^ who reported significant TEL in practicing physicians. Rather, the failure to find TEL in the present study highlights several important programmatic factors that should be considered when designing and implementing TEL within CME populations.

TEL literature has identified several practices that maximize the benefits of testing on learning:^18-20^

1. *Test format*. Overall, TEL is larger when individuals need to produce a response (e.g., short answer questions (SAQs)) rather than recognize the correct answer (e.g., MCQs).^21^ In the present study, the intervening and final test activities were composed entirely of MCQs, which may have contributed to the null findings in the present study. That being said, the present study used context-rich MCQs, which consist of a clinical case presentation and a lead in question. It has been argued that context-rich MCQs test the application of clinical and diagnostic knowledge as opposed to testing recollection of specific facts,^16,17^ and previous research has shown that context-rich MCQs produce testing effects comparable to those of SAQs in undergraduate medical studenta.^6^ However, the information included in the clinical scenarios was superficial (e.g., 55 yo male plumber vs. 59 yo male electrician), which may not be enough to engage effortful processing of information in practicing clinicians.
2. *Number and frequency of intervening tests*. Research suggests that TEL is enhanced when individuals are exposed to multiple tests separated by longer time intervals (e.g., days, weeks).^14,22^ The present study provided physicians with two tests, each separated by four weeks. The intention here was to provide participants with more than one testing activity that were separated by enough time to promote effortful retrieval. However, it is possible that two quizzes are not enough to promote learning within the CME contexts. In contrast, Larsen et al.^13^ had participants complete four tests, each separated by one-week, and was able to observe TEL within a CME context. More research is needed to determine the ideal number and spacing of intervening quizzes within CME populations.
3. *Feedback*. While TEL can be observed in the absence of feedback, the magnitude of TEL increases when feedback is provided to learners.^23^ In the present study, participants in the test group did not receive any feedback regarding their test performance. This was an intentional decision, as the goal of our study was to examine “direct” testing effects, that is, the effects of retrieval practice itself. Within the TEL literature, feedback is often considered an “indirect” testing effect, as feedback is thought to enhances learning by directing attention to areas in need of improvement.^24^ Anecdotally, several participants expressed interest in “finding out how they did,” suggesting that feedback may be important to this group of learners. Indeed, Larsen et al.^13^ provided participants with the feedback, which may help explain why these authors found evidence of TEL in their CME context while the present study did not. However, it is important to point out that Larsen et al. used identical questions for all assessment activities (e.g., pretest, practice quizzes, final test) and as a result, the provision of feedback may have artificially inflated their performance. More research is needed to disentangle different mechanisms of TEL (e.g., direct vs. indirect effects) within CME populations.

In the context of this literature, the null results demonstrate the importance of designing TEL activities to align with best practices identified in the literature, such as the provision of feedback and the format, number, and frequency of quizzes. Building on retrieval-practice theories, it is important to design TEL activities in a way that requires effortful retrieval practices.^5^ However, study designs that promote retrieval processes (e.g., frequent intervening quizzes, etc.) also come with their own logistical and administrative challenges, such as participant recruitment and attrition. A balance must be struck between research and practice implementing TEL within CME.

Finally, when designing TEL studies within CME contexts, it is important to acknowledge that practicing physicians bring with them a wide range of prior knowledge and experiences. This is one of the reasons randomization was used in the present study; however, after attrition, the group sizes were small (n=26 from test condition, n=23 from study condition), making it difficult to rule out potential differences between the two groups. For example, since the present study occurred over an 8-week period, some participants may have encountered clinical cases and patients related to the CME topics, which would have led to additional retrieval opportunities. Similarly, some participants may have been more knowledgeable in some CME topics relative to other participants. One solution would have been to provide participants with a pretest before the CME event; this would have allowed us to verify that participants’ knowledge on the topics were comparable across the two groups. More specifically, the use of a pretest/post-test design would have allowed us to statistically tease out potential differences associated with the intervention from differences associated with participants themselves. Such pretest designs may be particularly important in CME contexts that are attended by clinicians with a range of educational and clinical backgrounds.

To conclude, while the benefits of TEL are largely recognized among various educational settings, our study did not find this effect. These null results highlight the importance of designing TEL activities using best practices.^18^ More research is needed to examine the extent to which factors such as test format, spacing of quizzes, and provision of feedback moderate TEL within practicing clinicians.

## References

[1] Campbell C, Silver I, Sherbino J, Cate O Ten, Holmboe ES Competency-based continuing professional development. Med Teach. 2010;32(8):657–62. 20662577 10.3109/0142159X.2010.500708

[2] Davis D, Bordage G, Moores LK, Bennett N, Marinopoulos SS, Mazmanian PE, et al. The science of continuing medical education: Terms, tools, and gaps: Effectiveness of continuing medical education. Chest. 2009;135(3):8S–16S. 19265071 10.1378/chest.08-2513

[3] Larsen DP, Butler AC *Test-enhanced learning* In: Walsh K, editor. Oxford Textbook of Medical Education. Oxford: Oxford University Press; 2013 p. 443–52.

[4] Larsen DP, Butler AC, Roediger HL Test-enhanced learning in medical education. Med Educ. 2008;42(10):959–66. 18823514 10.1111/j.1365-2923.2008.03124.x

[5] Karpicke JD, Grimaldi PJ Retrieval-based learning: A perspective for enhancing meaningful learning. Educ Psychol Rev. 2012;24(3):401–18.

[6] Mcconnell MM, Young M, St-Onge C The benefits of testing for learning on later performance. Adv Heal Sci Educ. 2015;20(2):305–20. 10.1007/s10459-014-9529-1 24973998

[7] Schmidmaier R, Ebersbach R, Schiller M, Hege I, Holzer M, Fischer MR Using electronic flashcards to promote learning in medical students: Retesting versus restudying. Med Educ. 2011;45(11):1101–10. 21988625 10.1111/j.1365-2923.2011.04043.x

[8] Larsen DP, Butler AC, Lawson AL, Roediger HL The importance of seeing the patient: Test-enhanced learning with standardized patients and written tests improves clinical application of knowledge. Adv Heal Sci Educ. 2013 8;18(3):409–25. 10.1007/s10459-012-9379-7 22618856

[9] Larsen DP, Bulter AC, Roediger HL Repeated testing improves long-term retenton relative to repeated study: A randomised controlled trial. Med Educ. 2009;43:1174–81. 19930508 10.1111/j.1365-2923.2009.03518.x

[10] Dunlosky J, Rawson K, Marsh EJ, Nathan MJ, Willingham DT Improving students’ learning with effective learning techniques: Promising directions from cognitive and educational psychology. Psychol Sci Public Interes. 2013;14(1):4–58. 10.1177/1529100612453266 26173288

[11] Roediger HL Applying cognitivepsychology to education: Translational educational science. Psychol Sci Public Interes. 2013;14(1):1–3. 10.1177/1529100612454415 26173287

[12] McConnell M, Azzam K, Xenodemetropoulos T, Panju A Effectiveness of test-enhanced learning in continuing health sciences education: a randomized controlled trial. J Contin Educ Heal Prof. 2015;35(2):119–22. 10.1002/chp.21293 26115111

[13] Larsen D, Aung WY, Corboy J, Friedman D, Tilton A, Butler A The effects of repeated quizzing on long-term retention in AAN annual meeting courses. Neurology. 2015;84(7):748–54. 25609761 10.1212/WNL.0000000000001264 PMC4336103

[14] Rawson K, Dunloaky J When is practice testing most effective for improving the durability and efficiency of student learning? Educ Psychol Rev. 2012;24(3):419–35.

[15] Phelps D The effect of testing on student achievement. Int J Test. 2012;12(1):21–42.

[16] Medical Council of Canada Guidelines for the development of multiple-choice questions. Ottawa, ON; 2010.

[17] National Board of Medical Examiners Constructing written test questions for the basic and clinical sciences. Philadelphia, PA; 2002.

[18] Larsen D, Butler AC *Test-enhanced learning* In: Walsh K, editor. Oxford Textbook of Medical Education. Oxford; 2013 p. 443–52.

[19] Binks S Testing enhances learning: A review of the literature. J Prof Nurs, *epub ahead of print*. 10.1016/j.profnurs.2017.08.008 29929801

[20] Dunlosky J, Rawson K, Marsh EJ, Nathan MJ, Willingham DT Improving students’ learning with effective learning techniques promising directions from cognitive and educational psychology. Psychol Sci Public Interes. 2013;14(1):4–58. 10.1177/1529100612453266 26173288

[21] Kang SHK, Mcdermott KB, Iii HLR, Kang SHK, Mcdermott KB, Iii HLR, et al. Test format and corrective feedback modify the effect of testing on long-term retention testing on long-term retention. Eur J Cogn Psychol. 2007;19:528–58.

[22] Cepeda N, Coburn N, Rohrer D, Wixted J, MC M, Pashler H Spacing effects in learning: a temporal ridgeline of optimal retention. Psychol Sci. 2008;19:1095–1102. 19076480 10.1111/j.1467-9280.2008.02209.x

[23] Butler A, Karpicke J, Roediger H Correcting a metacognitive error: Feedback increases retention of low-confidence correct responses. J Exp Psychol Learn Mem Cogn. 2008;34(4):918–28. 18605878 10.1037/0278-7393.34.4.918

[24] Dochy F, Segers M, Gijbels D, Struyven K *Assessment engineering: Breaking down barriers between teaching and learning, and assessment* In: Boud D, Falchikov N, editors. Rethinking Assessment in Higher Education: Learning for the longer term. New York, NY: Routledge; 2007 p. 87–100.

